# Virome of red imported fire ants by metagenomic analysis in Guangdong, southern China

**DOI:** 10.3389/fmicb.2024.1479934

**Published:** 2024-11-08

**Authors:** Qiuxu Li, Yingjie Lian, Ketong Zhang, Jinchao Chen, Long Chen, Jiandong Wu, Yangyang Zhang, Minyi Chen, Weize Zhang, Mengke Lu, Jun Ma, Aiquan Bai

**Affiliations:** ^1^School of Animal Science and Technology, Foshan University, Foshan, China; ^2^College of Horticulture and Landscape Architecture, Zhongkai University of Agriculture and Engineering, Guangzhou, China

**Keywords:** virome, metagenomic analysis, RNAseq, red imported fire ant, virus

## Abstract

The red imported fire ant (RIFA) has made China its habitat for approximately 25 years, but few reports have described the species and amount of virus circulating in it. Researchers are currently exploring viruses associated with RIFAs as potential biological control agents against invasive ants. The present meta-transcriptome analysis revealed the virome of red imported fire ants in Guangdong, southern China, which included 17 viruses, including *Solenopsis invicta* virus 4-GD (SINV-4) and Guangdong Polycipiviridae ant virus 1 (GPAV1) in the *Polycipiviridae* family; *Solenopsis invicta* virus 1-GD (SINV-1), and Guangdong Dicistroviridae ant virus 2-3 (GDAV2-3) in the *Dicistroviridae* family; Guangdong Iflaviridae ant virus 4-9 (GIAV4-9) in the *Iflaviridae* family; Guangdong Parvoviridae ant virus 10 (GPAV10) in the *Parvoviridae* family; and Guangdong ant virus 11-15 (GAV11-15). A total of 15 novel viruses and 2 known viruses were identified in this study. These findings reveal the virome of red imported fire ants in Guangdong Province and present a different result from that of a similar study reported in the United States, providing more choices for potential classical biological control agents against red imported fire ants in China.

## 1 Introduction

The red imported fire ant (RIFA) is an invasive species originating from South America that exhibits wide adaptability, strong stress resistance, and a rapid reproductive rate it poses significant risks to both humans and animals because of its painful stings and bites, which can cause burns, severe shock, or even death (Kundrotas, 1993). This species can damage biodiversity and ecological stability by hunting other insects, small mammals, and birds (Vinson, 2013). Moreover, the production of crops, orchards, and horticultural plants is disrupted when red imported fire ant (RIFA) activity is located near farms (Wan and Yang, 2016; Xu et al., 2022).

*Solenopsis invicta,* colloquially known as the red imported fire ant (RIFA), is a species of ant that is notorious for its aggressive behavior and painful stings. It first invaded China in 1999 in the Taiwan Province and then quickly expanded to four cities, with a total area of more than 6,000 hectares (Lin et al., 2021). At the end of 2004, the RIFA was transmitted into mainland China and was discovered in Wuchuan city, Guangdong Province (Zeng et al., 2005). Presently, their distribution spans the southern and eastern provinces of China, such as Hainan, Guangdong, Guangxi, Fujian, Zhejiang, Guizhou, Yunnan, and Jiangxi (Bamisile et al., 2023; Wang et al., 2023). According to a Chinese article, between 1998 and 2020, a total of 862 human cases of ant stings were reported from hospitals, mostly from Quanzhou city of Fujian Province, Yangjiang city of Guangdong Province, and Guangzhou city of Guangdong Province. The clinical symptoms mainly include itch pain (100%), ruddy (99.2%), blain (98.8%), and pimple (59.0%), and severe symptoms such as systemic hypersusceptibility (0.8%) and shock (0.8%) rarely occur (Que Maoqi et al., 2021).

The exploration and utilization of biological control agents and populations have proven instrumental in the management and suppression of fire ants (Manfredini et al., 2016). Viruses are recognized as significant biological control agents against insect populations and have been studied extensively. High-throughput sequencing technology has emerged as a valuable tool for investigating viromes to diagnose unknown infectious diseases (Shi et al., 2016). Guangdong Province in China is acknowledged as a global hotspot for emerging zoonotic diseases and vector-borne diseases because of its unique climate, environment, and biodiversity (Wang et al., 2008; Allen et al., 2017). However, the viromes of RIFAs within Guangdong, southern China, remain inadequately understood.

## 2 Materials and methods

### 2.1 Sample collection and preprocessing

Nest soil from ants was collected in September 2022 from villages in Jieyang and Yunfu cities, Guangdong Province, China. After collection, the samples were transported to the laboratory within 24 h and stored at −80°C immediately. After being extracted from the nest soil, the ants were washed with 95% ethanol. Ant species were morphologically identified by an experienced technician and confirmed by sequencing the mitochondrial 16S ribosomal RNA (16S rRNA) gene. To better identify the viruses present in the samples, the ants from the two locations were synthesized into one group. After being washed with phosphate-buffered saline (PBS), the ants were homogenized in PBS, followed by centrifugation at 2,500 × g for 5 min at 4°C. The supernatants were collected for RNA extraction with TRIzol LS reagent (Invitrogen, Carlsbad, CA, United States).

### 2.2 Meta-transcriptomic and bioinformatics analyses

Meta-transcriptome sequencing was conducted as previously described (Shi et al., 2016). In brief, after the ribosomal RNA (rRNA) was removed, the RNA was fragmented, reverse-transcribed, and adapted, followed by paired-end (150 bp) sequencing on the Illumina HiSeq 2500 platform. All library preparation and sequencing were performed by Tianjin Novogene Bioinformatics Technology Co., Ltd., Tianjin, China.

The sequencing reads were adaptor-and quality-trimmed via the FASTP program, followed by *de novo* assembly via the Megahit program, and the resulting contigs were compared against the nr database via the diamond BLASTX program. The confirmed viral contigs with assembly overlaps, or from the same scaffold, were merged via the SeqMan program (version 7.1, DNAstar, Madison, WI, United States). Nested RT–PCR was conducted to fill gaps and verify the obtained sequence. The genomic terminus of the target viruses was determined via 5′/3′ RACE kits (TaKaRa, Dalian, China). The primers used are shown in Supplementary Table S2. The reads were mapped to the target contigs via Bowtie 2, and an integrated genomics viewer (IGV) was used to check for assembly faults to validate the assembly results.

### 2.3 Virus classification

The discovered viruses were classified on the basis of their nucleotide (nt) and amino acid (aa) identities. If the species demarcation criteria remain unclear within a genus, a novel viral species is defined if it holds less than 80% nt identity across the complete genome, or less than 90% aa identity of the RNA-dependent RNA polymerase (RdRp) domain with known viruses. All the novel viruses were named “Guangdong ant,” followed by common viral names according to their taxonomy. Viruses classified into established taxonomies were marked with “Guangdong (GD)” to distinguish them from other viral strains.

### 2.4 Phylogenetic analysis

Reference virus sequences were acquired from the GenBank database and examined via MAFFT v7.450 to determine the phylogenetic relationships of the identified viruses (Katoh et al., 2019). After testing with ProtTest 3.4, phylogenetic trees were constructed via the maximum-likelihood method in PhyML v3.0 with the Le and Gascuel substitution model for amino acid sequence analysis, which is based on a bootstrap value of 1,000 replicates (Darriba et al., 2011; Guindon et al., 2005).

## 3 Results

### 3.1 Viral diversity in ants in Guangdong

A total of 432 ants were collected from two distinct locations within Guangdong Province, China Jieyang (*n* = 324) and Yunfu (*n* = 108). Species identification was conducted through a comprehensive amalgamation of morphological scrutiny and 16S PCR techniques. Among the collected samples, 411 were unequivocally identified as *Solenopsis invicta*, and the remaining 21 were characterized as *Polyrhachis dives* and were excluded from this study (Supplementary Table S1; Supplementary Figure S1).

Ants were pooled into one group for RNA library construction and sequencing. After quality control and adapter trimming, a total of 85,900,504 paired-end clean reads were generated in these libraries, resulting in 79,054 viral reads, which accounted for 0.1% of the total RNA reads, and were assembled into 95 viral contigs. After alignment via BLAST, the viral contigs were ultimately annotated to 17 viruses from the viral families *Polycipiviridae*, *Dicistroviridae*, *Iflaviridae*, *Parvoviridae*, and unclassified families. The raw data and obtained virus sequences have been uploaded to NCBI with Bioproject Accessions: PRJNA1061181; Biosample Accessions: SAMN39259552; and SRA accessions: SRR27458542.

### 3.2 Viral genome organization and phylogenetic characterization

The genomes of 17 viruses, including 15 previously unknown viruses, were obtained through contig-based PCR and rapid amplification of cDNA ends (RACE). The complete genomes of 14 viruses were obtained and named Guangdong Dicistroviridae ant virus 2–3 (GDAV2-3), Guangdong Iflaviridae ant virus 4–9 (GIAV4-9), Guangdong Parvoviridae ant virus 10 (GPAV10), Guangdong ant virus 12,13,15 (GAV12,13,15), *Solenopsis invicta* virus 1-GD (SINV1 GD) and *Solenopsis invicta* virus 4-GD (SINV4 GD). Additionally, three viruses with partial sequences were identified and named Guangdong Polycipiviridae ant virus 1 (GPAV1), Guangdong ant virus 11 (GAV11) and Guangdong ant virus 14 (GAV14) (Table 1).

**Table 1:** Information on the viral genome obtained in this study.

Virus name	Virus abbreviation	Accession number	Viral family	Genetically closest virus	GenBank accession number of genetically closest virus	Length of the segment (bp)	Identity with relative virus (nt, %)	Protein*	Identity with relative virus (aa, %)
*Solenopsis invicta* virus 4-GD	SINV4 GD	PP104790	*Polycipiviridae*	*Solenopsis invicta* virus 4	NC 035455	12,102	90.8	Putative capsid protein	99.3
								Hypothetical protein	96.4
								Putative capsid protein	98.88
								Putative capsid protein	95.28
								RNA-dependent RNA polymerase	96.13
Guangdong Polycipiviridae ant virus 1	GPAV1	PP104791	*Polycipiviridae*	Polycipiviridae sp.	MZ679315	11,474	83.9	Putative RNA-dependent RNA polymerase	85.77
								Putative capsid protein 1	98.2
								Putative capsid protein 2	94.83
								Putative capsid protein 3	94.59
								Hypothetical protein	95.82
*Solenopsis invicta* virus 1-GD	SINV1 GD	PP104795	*Dicistroviridae*	*Solenopsis invicta* virus 1	NC 006559	8,026	94.2	Nonstructural polyprotein	98.21
								Contains capsid proteins; structural polyprotein	99.03
Guangdong Dicistroviridae ant virus 2	GDAV2	PP104803	*Dicistroviridae*	Beihai picorna-like virus 76	KX883903	5,649	62	Hypothetical protein	61.66
Guangdong Dicistroviridae ant virus 3	GDAV3	PP104794	*Dicistroviridae*	Wuhan arthropod virus 2 strain WHSFII47608	KX884287	9,875	45.9	Hypothetical protein 1	64.87
								Hypothetical protein 2	66.94
Guangdong Iflaviridae ant virus 4	GIAV4	PP104799	*Iflaviridae*	Infectious flacherie virus	NC 003781	9,650	60.8	Polyprotein	58.95
Guangdong Iflaviridae ant virus 5	GIAV5	PP104801	*Iflaviridae*	King virus	NC 031749	10,193	58.2	Polyprotein	44.01
Guangdong Iflaviridae ant virus 6	GIAV6	PP104798	*Iflaviridae*	Opsiphanes invirae iflavirus 1	NC 027917	9,855	47	Polyprotein	51.2
Guangdong Iflaviridae ant virus 7	GIAV7	PP104802	*Iflaviridae*	*Lampyris noctiluca* iflavirus 1	MH620811	10,339	46.1	Putative polyprotein	34.94
								Putative polyprotein	33.93
Guangdong Iflaviridae ant virus 8	GIAV8	PP104793	*Iflaviridae*	Moku virus isolate Big Island	NC 031338	10,056	36.0	Polyprotein	56.05
Guangdong Iflaviridae ant virus 9	GIAV9	PP104792	*Iflaviridae*	Moku virus isolate Big Island	NC 031338	10,056	36.3	Polyprotein	54.78
Guangdong Parvoviridae ant virus 10	GPAV10	PP104806	*Parvoviridae*	*Periplaneta fuliginosa* densovirus	NC 000936	5,454	65.7	Structural protein	53.04
								Structural protein	56.8
								Nonstructural protein	67.85
								Hypothetical protein PfdVgp6	61.51
								Hypothetical protein PfdVgp6	26.73
Guangdong ant virus 11	GAV11	PP104805	unclassified *Dicistroviridae*	Dicistroviridae sp. 1	MZ394716	6,015	49	Putative structural protein	90.98
Guangdong ant virus 12	GAV12	PP104800	unclassified RNA viruses	Beihai picorna-like virus 70	NC 032249	9,214	44	Hypothetical protein	31.95
Guangdong ant virus 13	GAV13	PP104797	unclassified viruses	Vespa velutina associated acypi-like virus	MN565042	9,900	46.3	P1 protein	98.88
								P2 protein	98.04
Guangdong ant virus 14	GAV14	PP104804	unclassified *Picornavirales*	*Lycopersicon esculentum* picorna-like virus	MN832456	9,157	45.3	Hypothetical protein	21.8
Guangdong ant virus 15	GAV15	PP104796	unclassified RNA viruses	Changjiang crawfish virus 6	NC 032801	9,743	44.9	Glycoprotein	36.5

### 3.3 *Polycipiviridae*: SINV4 GD, GPAV1

Metagenomic analysis revealed that approximately 2.2% (1,722/79,054) and 0.2% (195/79,054) of the total reads mapped to the genomes of SINV4 GD and GAV7, respectively (Supplementary Table S3). We obtained two complete viral genomes belonging to *Polycipiviridae* and designated them SINV4 GD and GAV7.

**Figure 1:**
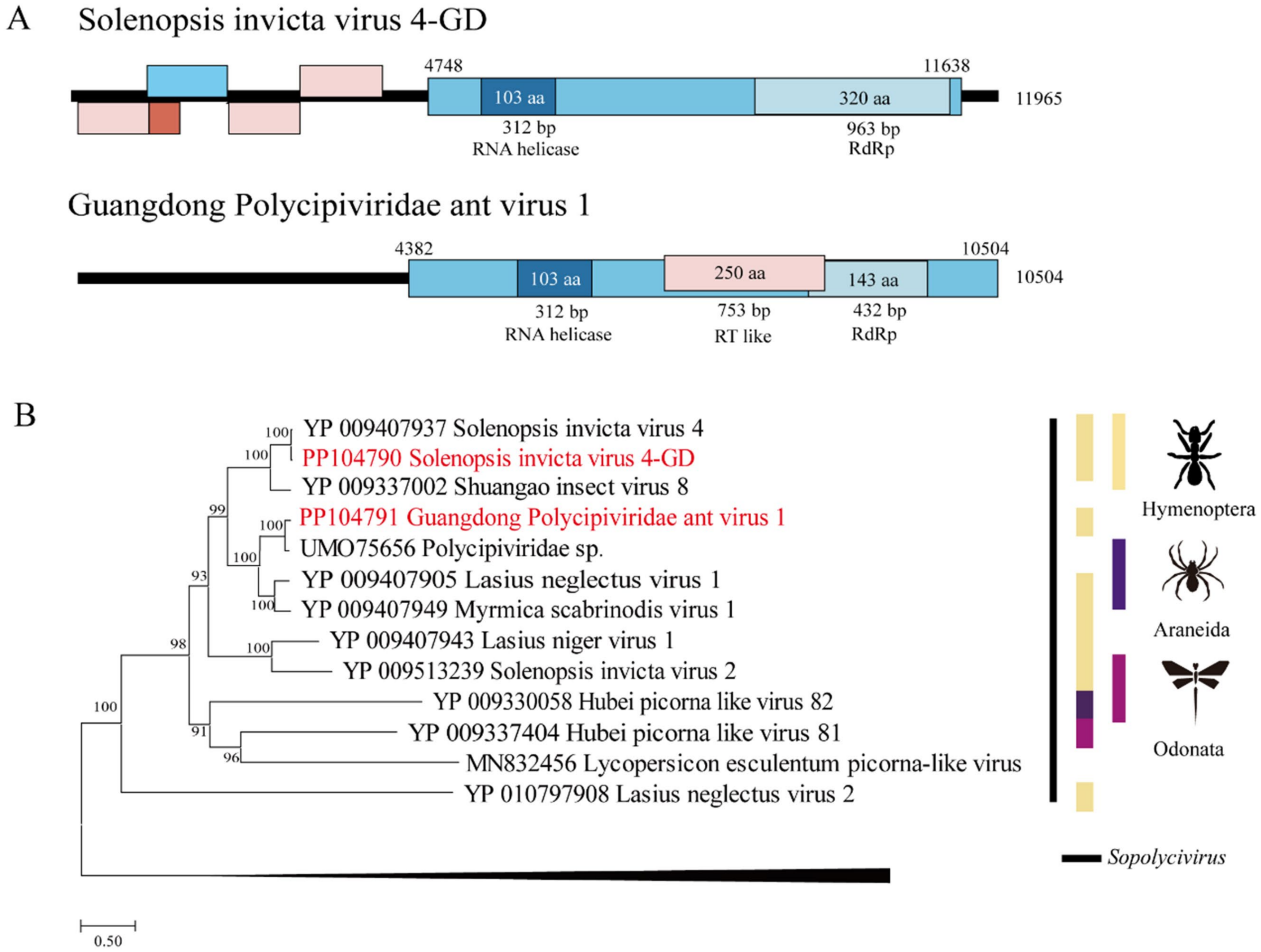
Genome characterization and phylogenetic analysis of *Polycipiviridae.* (A) Genome organization and putative coding regions of *Solenopsis invicta* virus 4-GD (SINV4) and Guangdong Polycipiviridae ant virus 1 (GPAV1). Both viral genomes include RNA helicases and RNA-dependent RNA polymerase (RdRp). (B) Phylogenetic analyses of *Polycipiviridae*. Phylogenetic trees were constructed on the basis of the RdRp amino acid sequences of representative viruses in the family *Polycipiviridae*. The viruses obtained in this study are highlighted in red. The scale bar at the bottom indicates the amino acid substitutions per site. The vertical color bars at the right of the tree indicate the order of the virus host. The vertical black line at the right of the tree indicates the virus genus.

The genome of SINV4 GD includes five ORFs (Figure 1) encoding the RdRp and RNA helicase. The length of the SINV4 GD is 11,965 bp, and it shares high identity (96.1% of the RdRp aa sequence, 90.8% of the complete nt sequence) with *Solenopsis invicta* virus 4, which was identified in the United States (Olendraite et al., 2017). The GPAV1 genome is 10,504 bp in length, and its RdRp gene shares 83.9 and 85.8% nt and deduced aa sequence identity with the closest viral strain, Polycipiviridae sp., which was identified in China (Chen et al., 2022). Phylogenetically, both SINV4 GD and GPAV1 identified in this study were grouped into the *Polycipiviridae* family.

### 3.4 *Dicistroviridae*: SINV1 GD, GDAV2, and GDAV3

In total, 28,419 reads (35.9%; 28,419/79,054) were mapped to viruses in the *Dicistroviridae* family. A total of 0.3% of the reads (209/79,054) in the pools were matched with SINV1 GD, 0.2% of the reads (261/79,054) were mapped with GDAV2, and 35.4% of the reads (27,994/79,054) were matched with GDAV3 (Supplementary Table S4).

The length of the SINV1 GD was 9,944 bp, with high identity (98.2% of the RdRp aa sequence and 94.2% of the complete nt sequence) to *Solenopsis invicta* virus 1, which was identified in the United States (Valles et al., 2013). The length of GDAV3 is 10,144 bp, and its RdRp gene shares 45.9 and 66.9% nt and deduced aa sequence identity, respectively, with the Wuhan arthropod virus 2 strain WHSFII47608 (Shi et al., 2016). The length of GDAV2 was 7,418 bp, and its RdRp gene shares 62.0 and 61.7% nt and deduced aa sequence identity with the strain Beihai picorna-like virus 76 (Supplementary Table S4).

**Figure 2:**
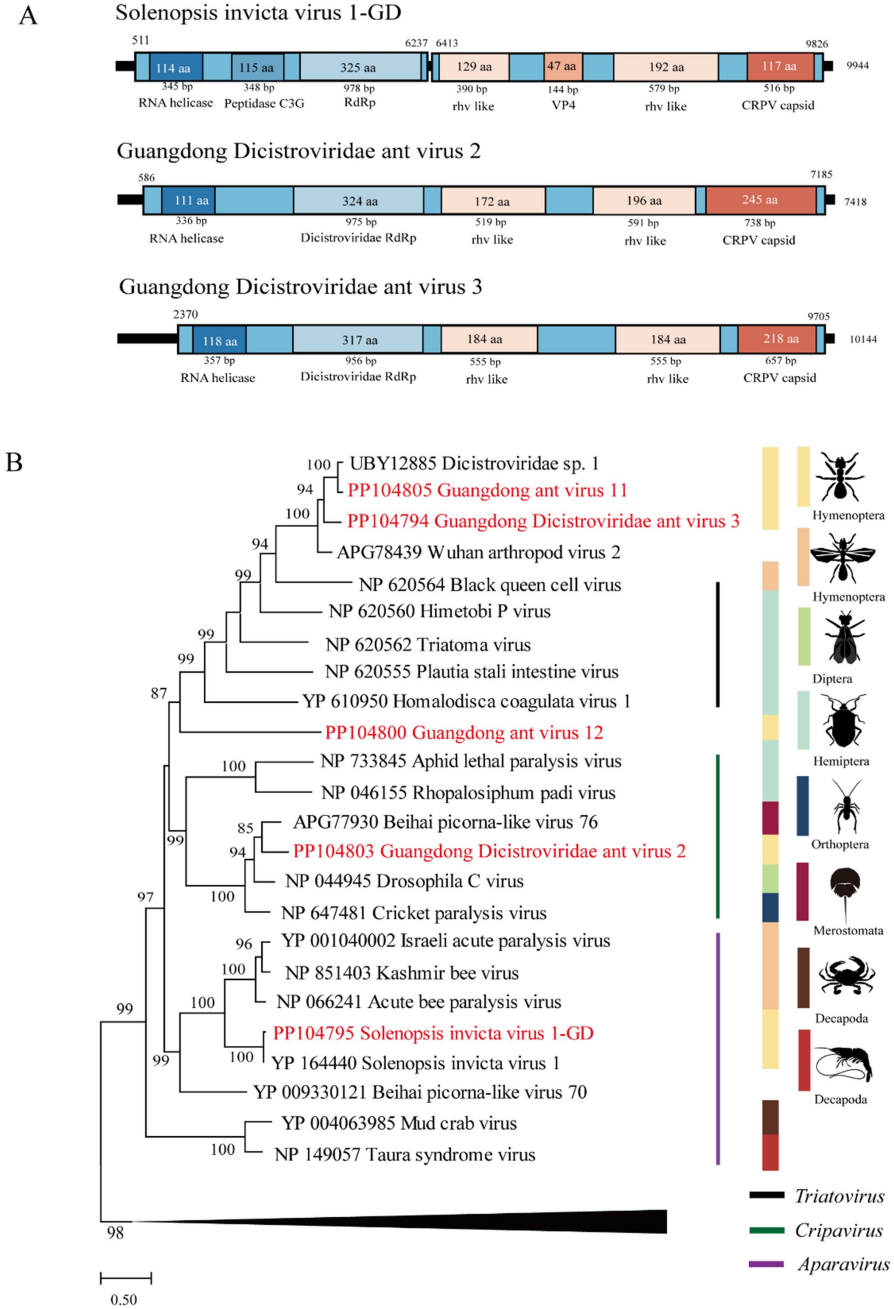
The genome structure and phylogenetic analysis of *Dicistroviridae*. (A) Genome organization and putative coding regions of *Solenopsis invicta* virus 1-GD (SINV1), Guangdong Dicistroviridae ant virus 2 (GDAV2), and Guangdong Dicistroviridae ant virus 3 (GDAV3). The viral genome contains segments encoding the RdRp and RNA helicases. (B) Phylogenetic analyses of *Dicistroviridae*. Phylogenetic trees were constructed on the basis of the RdRp protein sequences of representative viruses in the family *Dicistroviridae*. The viruses obtained in this study are highlighted in red. The scale bar at the bottom indicates the amino acid substitutions per site. The vertical color bars at the right of the tree indicate the order of the virus host. The vertical color lines at the right of the tree indicate the virus genera.

Phylogenetically, the SINV1 GD, GDAV2, and GDAV3 genes identified in this study were grouped within the *Dicistroviridae* clade (Figure 2).

### 3.5 *Iflaviridae*: GIAV4-9

Metagenomic analysis revealed that a total of 6.8% (5,408/79,054) of the viral reads mapped to the genomes of the iflavirus, which included 1.4% (1,114/79,054) GIAV4, 0.4% (345/79,054) GIAV5, 0.2% (172/79,054) GIAV6, 0.6% (506/79,054) GIAV7, 2.2% (1,743/79,054) GIAV8, and 1.9% GIAV9 (1,528/79,054) reads.

The length of GIAV4 is 9,727 bp, and its RdRp gene shares 61 and 59% nt and deduced aa sequence identity with the infectious flounder virus strain (Isawa et al., 1998). The length of GIAV5 was 8,459 bp, and its RdRp gene shares 58.2 and 44% nt and deduced aa sequence identity with the strain King virus. The length of GIAV6 is 9,827 bp, and its RdRp gene shares 47 and 51.2% nt and deduced aa sequence identity with the strain Opsiphanes invirae iflavirus 1 (Silva et al., 2015). The length of GIAV7 is 7,799 bp, and the RdRp gene of GIAV7 is 46.1% nucleotide and has amino acid sequence identities with the *Lampyris noctiluca* iflavirus 1 strain, with amino acid identities ranging from 33.9 to 34.9%. Compared with those of the Moku virus isolate Big Island, the lengths of GIAV8 and GIAV9 are 10,320 bp and 10,336 bp, indicating nucleotide identities of 36 and 36.3%, respectively, and amino acid identities of 56.1 and 54.8%, respectively, in the RdRp region (Supplementary Table S5) (Mordecai et al., 2016).

**Figure 3:**
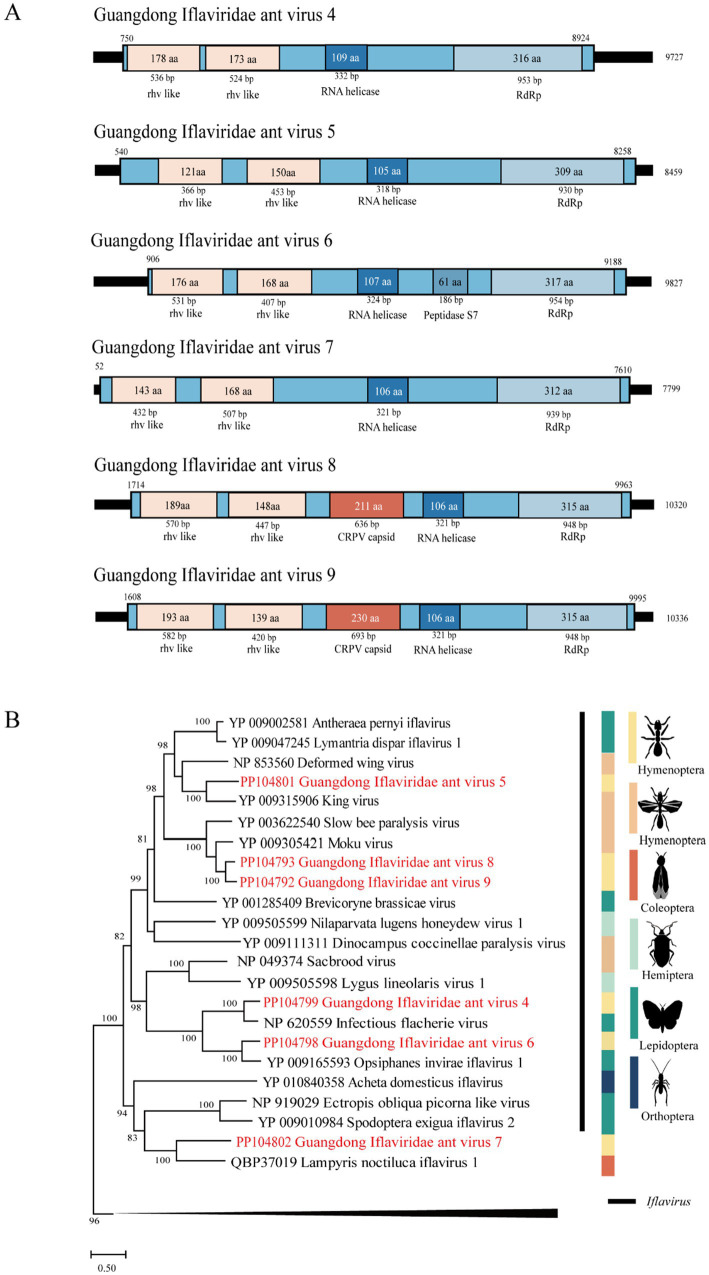
The genome structure and phylogenetic analysis of *Iflaviridae*. (A) Genome organization and putative coding regions of Guangdong Iflaviridae ant virus 4–9 (GIAV4-9). The viral genome contains segments that encode RdRp, rhv-like, and RNA helicases. (B) Phylogenetic analyses of members of the family *Iflaviridae*. Phylogenetic trees were constructed on the basis of the RdRp protein sequences of representative viruses in the family *Iflaviridae*. The viruses obtained in this study are highlighted in red. The scale bar at the bottom indicates the amino acid substitutions per site. The vertical color bars at the right of the tree indicate the order of the virus host. The vertical black line at the right of the tree indicates the virus genus.

Phylogenetically, the six viruses identified in this study were grouped into the *Iflaviridae* clade (Figure 3).

### 3.6 *Parvoviridae*: GPAV10

**Figure 4:**
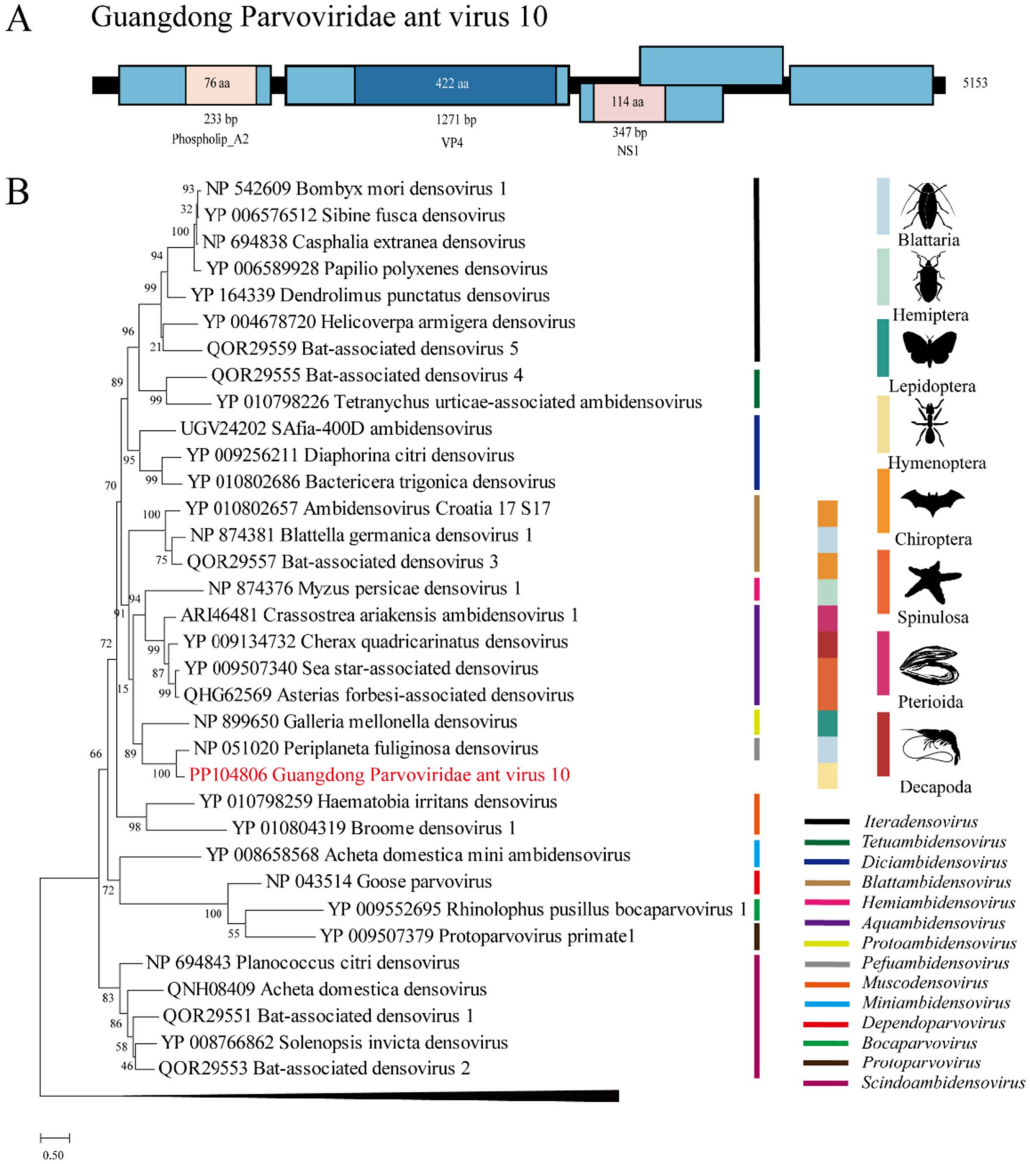
The genome structure and phylogenetic analysis of *Parvoviridae*. (A) Genome organization and putative coding regions of Guangdong ant Parvoviridae virus 8 (GPAV10). The viral genome contains segments that encode NS1, VP4, and phospholip A2. (B) Phylogenetic analyses of *Parvoviridae*. Phylogenetic trees were constructed on the basis of the NS1 protein sequences of representative viruses in the *Parvoviridae* family. The viruses obtained in this study are highlighted in red. The scale bar at the bottom indicates the amino acid substitutions per site. The vertical color bars at the right of the tree indicate the order of the virus host. The vertical color lines at the right of the tree indicate the virus genera.

Approximately 44.4% of the reads (35,116/79,054) were mapped to the genome of Guangdong Parvoviridae ant virus 8 (GPAV10), which is located in the genus *Pefuambidensovirus* in the phylogenetic tree. We designated this virus GPAV10, whose genome shares 65.7% and 26.7 ~ 67.9% nt and deduced aa sequence identity with *Periplaneta fuliginosa* densovirus (Guo et al., 2000) (Figure 4).

### 3.7 Unclassified viruses: GAV11-15

The length of GAV11 is 5,249 bp, and its RdRp gene shares 49 and 91% nt and deduced aa sequence identity with the strain Dicistroviridae sp. 1. The length of GAV14 is 7,345 bp, and its genome shares 45.3% nt identity with that of the *Lycopersicon esculentum* picorna-like virus. The length of GAV12 is 9,640 bp, and its RdRp gene shares 44% nt and 32% nt and deduced aa sequence identity with the strain Beihai picorna-like virus 70 (Shi et al., 2016). For GAV11, GAV14 and GAV12 may only have partial RdRp information or high diversity with their closest viruses; thus, these viruses were deemed unclassified viruses in this study.

Vespa velutina-associated acypi-like virus, first discovered in the Asian yellow-legged hornet Vespa velutina nigrithorax, is an unclassified virus (Dalmon et al., 2019). In this study, a total of 344 (0.4%, 344/79,054) reads were mapped to the Vespa velutina-associated acypi-like virus. We named this virus Guangdong ant virus 13 (GAV13), which has 9,860 nt and shares 46.3% and 98 ~ 98.9% nt and deduced aa sequence similarity with the Vespa velutina-associated acypi-like virus.

Changjiang crawfish virus 6 is an unclassified RNA virus (Shi et al., 2016). In this study, a total of 3,214 (4.1%, 3,214/79,054) reads were mapped with the Changjiang crawfish virus 6. We named this virus Guangdong ant virus 15 (GAV15), whose genome is 9,901 nt long and shares 44.9 and 36.5% nt and whose aa sequence similarity is with that of the Changjiang crawfish virus 6.

## 4 Discussion

RIFA originates from South America, but studies indicate that the United States may be the main source of its invasion in China (Ascunce et al., 2011). This species can be transported to new areas through human transport, such as ships, trains and trucks (Wan and Yang, 2016). This study reported the results of a metagenomic analysis of RNA viruses associated with RIFA in Guangdong, southern China, revealing high viral diversity within this region, which is different from the results of a similar study reported in the United States (Valles and Rivers, 2019) (Supplementary Table S6). Seventeen different viruses belonging to four families, namely, *Polycipiviridae*, *Dicistroviridae*, *Iflaviridae*, *Parvoviridae*, and unclassified families, were identified.

*Polycipiviridae* is a family of picorna-like viruses with nonsegmented, linear, positive-sense RNA genomes of approximately 10 ~ 12 kb. All the members of the species within the family have been derived from arthropods, mostly from ants (Olendraite et al., 2017). The *Solenopsis invicta* virus 4 found in Guangdong Province is closely related to the virus found in the United States, and this result is consistent with the previous conclusion that RIFAs in China are from the United States (Ascunce et al., 2011). We also found that a novel virus, GPAV1, clustered in the *Polycipiviridae* family, and formed a distinct group from other polycipiviruses discovered in ants in the phylogenetic tree.

*Dicistroviridae* is a family of small nonenveloped viruses with RNA genomes of approximately 8 ~ 10 kilobases (Valles et al., 2017a). Two novel viruses, GDAV2 and GDAV3, were identified and phylogenetically clustered in the *Dicistroviridae* family. The genome of *Solenopsis invicta* virus 1 identified in this study is highly similar to that of strains reported in the United States, and is considered a potential tool for preventing the expansion of RIFAs (Hashimoto and Valles, 2007; Valles and Rivers, 2019).

*Iflaviridae* is a family of small nonenveloped viruses with RNA genomes of approximately 9 ~ 11 kilobases in length encoding a single polyprotein. Currently, iflaviruses are identified mainly from arthropods and primarily from insects (Valles et al., 2017b). In this study, six novel viruses belonging to this family were identified in Guangdong, China, suggesting that diverse iflaviruses circulate in RIFAs in this area.

*Parvoviridae* is a family of nonenveloped, round, icosahedral symmetric viruses with an approximately 4-to 6-kb-long single-stranded DNA genome (Cotmore et al., 2019). In the ICTV classification, *Parvoviridae* is divided into two subfamilies: *Parvovirinae*, which infect mammals and birds, and *Densovirinae*, which infect arthropods. In this study, we found that a novel virus, GPAV10, which is phylogenetically located in *Densovirinae*, occupied approximately half of the viral reads in the pool of RIFAs. This result may indicate that GPAV10 is highly infectious to RIFAs.

## 5 Conclusion

This study investigated the virome of red imported fire ants in Guangdong, southern China, and detected 17 viruses, including 14 viruses with complete genomes and 3 viruses with partial genomes. A total of 15 novel viruses and 2 known viruses were identified in this study. These findings provide information on viruses circulating in red imported fire ants in Guangdong Province and offer more options for potential classical biological control agents against red imported fire ants.

## Data Availability

The datasets presented in this study can be found in online repositories. The names of the repository/repositories and accession number(s) can be found in the article/Supplementary material.
