# Supplementary material for: Virome of red imported fire ants by metagenomic analysis in Guangdong, southern China
Source: Front Microbiol. 2024 Nov 8;15:1479934. doi: 10.3389/fmicb.2024.1479934 (PMC11582037; doi:10.3389/fmicb.2024.1479934)
Supplement: Supplementary file 1 [file Data_Sheet_1.pdf]

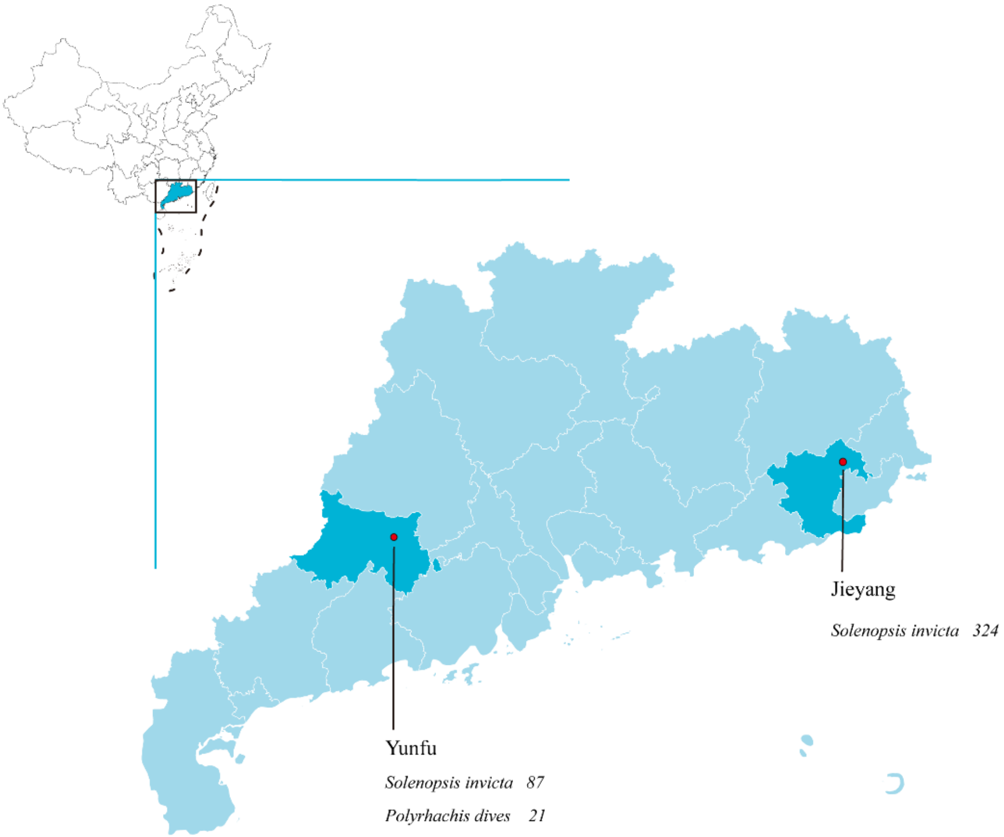

Supplementary Fig. S1. Ant samples were collected in the country area from Yunfu city and Jieyang city. The sampling sites in Guangdong Province are marked by red dots.

Supplementary Table S1. Ants collected in Guangdong Province, southern China

Regions	Ant species	No. of collected ticks
Jieyang	*Solenopsis invicta*	324
Yunfu	*Solenopsis invicta*	87
	Polyrhachis dives	21
Total		432

Supplementary Table S2. List of primers used for RT–PCR and 5'/3' RACE

Virus	Primers	Position (bp)[1]	Sequence (5'-3')	Amplicon (bp)
SINV1 GD	Detection			
	SINV1-F1	1316	CTTCAGAATTAATAGATTTA	
	SINV1-R1	2284	TTTTGTCCTTGATAATTATC	988
	SINV1-F2	1663	GCATTTGTTGGATTTATAC	
	SINV1-R2	1949	TCTTCAACTTCCAAGATTG	305
	Amplification			
	SINV1-Pept-F1	3796	ACTATGGTAAAACCTAAAGTTAAG	
	SINV1-Pept-R1	5683	GAAGGCCTTATGTTCTTCAAAACA	1911
	SINV1-Pept-F2	4269	TGCAGAAGTTAATTTACCTT	
	SINV1-Pept-R2	5119	ACCTTTTGCATCACGTCAGG	870
	SINV1-rhv_like-F1	5871	ATTTACATATACAGACGAAACAAA	
	SINV1-rhv_like-R1	8493	CATATTCACAAGGAACAGTATCCA	2646
	SINV1-rhv_like-F2	6712	TGAATCAAACGATAACCAC	
	SINV1-rhv_like-R2	7930	GTCAGTGATCAAACCCTGT	1237
	SIV 1-5’-GSP-R1	1050	TGTCTCCGCACTGTTCCAGATGTCTT	
	UPM			1050
	SIV 1-5’-GSP-R2	1008	ACTATGGCCAGCAGACATAAGATGCACG	
	UPM short		CTAATACGACTCACTATAGGGC	1008
	SIV 1-3’-GSP-F1	9004	CGAGTTCGAGCGAACACCAGGTTTGACC	
	UPM			940
	SIV 1-3’-GSP-F2	9262	TGCAGTAATGGGCAAAATTGCGGG	
	UPM short		CTAATACGACTCACTATAGGGC	682
SINV4 GD	Detection			
	SINV4-F1	9365	GCGGAATACCAACCAAGT	
	SINV4-R1	10420	GAGAAGCACGACAATCAAC	1056
	SINV4-F2	9614	CCGATTATGAGCATCTTGAC	
	SINV4-R2	10377	TCCAGTGACGCATTCTTC	764
GPAV1	Detection			
	GPAV1-F1	3385	GCTTCCACTCCATATCTTCT	
	GPAV1-R1	4473	CTATGTTCACCGCACTATTAG	1089
	GPAV1-F2	3846	TGTGTTATACGCTTGTCTTC	
	GPAV1-R2	4381	GCTGGAATGGTTATTGGAAT	536
GPAV10	Detection			
	GPAV10-F1	1525	ATGGTGCTGAAGTAGTTGAA	
	GPAV10-R1	2854	AATGTTGCTTGGAGGTGAT	1330
	GPAV10-F2	1963	CACTTGTTGGCGAACCTA	
	GPAV10-R2	2475	CAGTACCAGCAGATAGATAATG	513
GDAV2	Detection			
	GDAV2-F1	826	CGATCTTGCAATACTTTTCCCC	
	GDAV2-R1	969	AGCATGCTCACCAATTCTATTA	165
	GDAV2-F2	864	GGCTTTACATGACAAAAATA	
	GDAV2-R2	955	ATTATAAAAAGCTTGAGG	109
	Amplification			

	GDAV2-1-F1	845	CCCAGCATCTCCATATGG	
	GDAV2-1-R1	2131	ACTTCACTTGCACACAATT	1305
	GDAV2-1-F2	1131	TTGTGATAAGAAGTGGGT	
	GDAV2-1-R2	1600	AATTTAATGTCAAGCCAAC	488
	GDAV2-2-F1	3782	TGCGGCATTTTCGAAAGTA	
	GDAV2-2-R1	6352	TTATTGGACAATTCGTTAC	2554
	GDAV2-2-F2	4593	TCTTCCTGTATCTTATTT	
	GDAV2-2-R2	5495	TTCGATTCACAAGGAAGGC	921
	GDAV2 1-3'-GSP-F1	6608	TTTCTGTATTACAGGCTGCCACCACC	
	UPM			810
	GDAV2 1-3'-GSP-F2	6769	GCGTGGACCGTGCGGATGTTTACGTCCT	
	UPM short		CTAATACGACTCACTATAGGGC	649
GIAV4	Detection			
	GIAV4-F1	1137	AGGAGAAGTCACTGGAGAA	
	GIAV4-R1	2241	CGGATTCAATGGATTCAACA	1105
	GIAV4-F2	1303	GGACCAGAAGGACCGATA	
	GIAV4-R2	1807	GTAGATAACGACTGTCATAAGG	505
GIAV5	Detection			
	GIAV5-F1	1769	TCAACCAAATATAGGTTTATG	
	GIAV5-R1	2270	TTTTCTTTGGGAACCCATGGT	522
	GIAV5-F2	1943	TCCAAAATTCTTTAATAAAAC	
	GIAV5-R2	2186	GCGTCTGTAATTATTTTTTGT	264
	Amplification			
	GIAV5-1-F1	1867	ATTATTTTAGAGTAAATGAT	
	GIAV5-1-R1	4104	ATACTTTTAAGTAACTCAAC	2257
	GIAV5-1-F2	2506	GATTAGATCTCAATGTAGGTA	
	GIAV5-1-R2	3277	GTTAAGCCTATTTGTACTATTT	793
	GIAV5-5'-GSP-R1	1039	GGTAATGGTGCTCTTGGATCTGGAGGAT	
	UPM			
	GIAV5-5'-GSP-R2	1010	CACGATTGGGATCCGTAAAAGATCTC	
	UPM short		CTAATACGACTCACTATAGGGC	
	GIAV5-3'-GSP-F1	7364	CCCTATTGAGAAATGTTCTATTCCGGGC	
	UPM			1095
	GIAV5-3'-GSP-F2	7527	GAGTGGGATATACTTACCCGCAAGTTAT	
	UPM short		CTAATACGACTCACTATAGGGC	936
GAV11	Detection			
	GAV11-F1	185	AGAGGTATTGAGCGTATTGT	
	GAV11-R1	936	ACTAGCACACTGAGGATTC	752
	GAV11-F2	194	GAGCGTATTGTTGAATGGAT	
	GAV11-R2	558	TATAGTGACTGGAGGAGAATAG	365
GIAV6	Detection			
	GIAV6-F1	3944	TTTCCAATGTTGGTTTGACGAATT	
	GIAV6-R1	4655	AACGTCATGAAGAATATCATTCAG	735
	GIAV6-F2	4211	AGTACAAATCCAGTGTTGATTCTC	
	GIAV6-R2	4445	CTGAAAGGTATCAACAGTTGCCAG	347
	Amplification			

	GIAV6-1-F1	1999	ACACTTTCCCATGCTTCATTAGCA	
	GIAV6-1-R1	4848	TAAGGTGTGTTGGTCTAATACCGCT	2874
	GIAV6-1-F2	2647	GTTTCCTTTTGTTCAGATTGC	
	GIAV6-1-R2	3718	GCCATATTCTGTATTAAATGGTGG	1095
	GIAV6-2-F1	5668	GTTTTCATGAACACATTCTT	
	GIAV6-2-R1	8279	TCAGCTTTAAAAGATGTTGTCG	2633
	GIAV6-2-F2	6467	GCAAAGCAAACAAATCACTT	
	GIAV6-2-R2	7663	AATGGAGATCCTGGTGAAGT	1216
	GIAV6-5'-GSP-R1	923	GGTAAAGCTATAGCATCAGCATTCCTAG	
	UPM			923
	GIAV6-5'-GSP-R2	888	CGTAGTAACGGATGAAACAAAGGAC	
	UPM short		CTAATACGACTCACTATAGGGC	888
	GIAV6-3'-GSP-F1	8921	CAGCAATTGGTTCCAATTTCGCTAATG	
	UPM			905
	GIAV6-3'-GSP-F2	9036	CTCTCCAACTTGCATAACTTCAGTTTC	
	UPM short		CTAATACGACTCACTATAGGGC	790
GAV13	Detection			
	GAV13-F1	6551	CCCACTGCTGTCATAGCAAGTAA	
	GAV13-R1	7263	TAAAACACGACCAGAAATTGTTC	735
	GAV13-F2	6673	ACATTCAGCTACGAAAGATCTGGC	
	GAV13-R2	6989	CCGCTGTTTCCCAGGGAAAACATT	340
	Amplification			
	GAV13-1-F1	5064	GTAGCGCAAAACTACTAGGTTC	
	GAV13-1-R1	8093	GACCGAAAACAAAATAGAGTA	3050
	GAV13-1-F2	5827	AACGTTAGTGTAGCACTCTGGTA	
	GAV13-1-R2	7428	AACATCATCATTCGACCACCAC	1623
	GAV13-3'-GSP-F1	8308	CACCTTAAGGTGACATGGTGCGGTCTTA	
	UPM			1552
	GAV13-3'-GSP-F2	8385	TGGTGAATTTTGGGACGAATGGGTCATC	
	UPM short		CTAATACGACTCACTATAGGGC	1475
GIAV7	Detection			
	GIAV7-F1	6616	AGATTCGATGTATCAGTCTC	
	GIAV7-R1	6934	GCTACTACCTCCCCCCACCA	338
	GIAV7-F2	6699	AACTTTAAAAACAATTTCAC	
	GIAV7-R2	6880	GATATCCATTCAATTGCAGA	201
	Amplification			
	GIAV7-1-F1	5205	CAAAGATAAAACAGGCCAAC	
	GIAV7-1-R1	7135	CTCATGCAGCTTCAGGCTT	1949
	GIAV7-1-F2	5724	ATATCTATTTGGGGACAAAG	
	GIAV7-1-R2	6773	ATTTTGTACCCATCCTTTAG	1069
	GIAV7-3'-GSP-F1	7159	CCAGCCACCTCAGACACACCAGGGATGA	
	UPM			640
	GIAV7-3'-GSP-F2	7246	ATACCTTGAGGTTCAAGGGGGGCAAC	
	UPM short		CTAATACGACTCACTATAGGGC	553
GDAV3	Detection			
	GDAV3-F1	4619	ATGTCCAGAAGGTAGAGAATG	

	GDAV3-R1	6033	GCAGTATGTCACGAGTAGAT	1415
	GDAV3-F2	5257	CACCATCAATTCCACAACAA	
	GDAV3-R2	5959	AAGGCGTGTCTGCTTATC	703
GAV14	Detection			
	GAV14-F1	4588	GGTCCTATGTCAGTTCTAGTT	
	GAV14-R1	5907	GAGTGTTCTATATCCAGTTAGC	1320
	GAV14-F2	4697	AAGCGTCTAACTCAGAAGG	778
	GAV14-R2	5474	AACCAGGATAAGCCAACAG	
GAV15	Detection			
	GAV15-F1	5847	ATAATGTGAAGTGCGTAAGC	
	GAV15-R1	7636	GGAATAGCCAGAAGGATAGG	1790
	GAV15-F2	6592	AACGATTAGGAGGAGTTGAG	
	GAV15-R2	7337	TAGAGCAGGTGTGGTGAA	746
GIAV6	Detection			
	GIAV6-F1	3873	CCAACACAACTCTCAACTATG	
	GIAV6-R1	4742	CCACGGTTATGCTACACTT	870
	GIAV6-F2	4114	TGGCAAGTGGACTATCTAAC	
	GIAV6-R2	4620	GTTCTCTATTGACCGATGGA	507
GIAV8	Detection			
	GIAV8-F1	942	TCAGGAGCATCATTCATTCT	
	GIAV8-R1	1783	GAGTCAGGTAAGAGTAAGATTG	842
	GIAV8-F2	1126	GAGCATCTACATAAGCACTAC	
	GIAV8-R2	1695	ATCCTCGTCAACCTATTGG	570
GIAV9	Detection			
	GIAV9-F1	1070	ATAGTTGGCGAGGTTAGTC	
	GIAV9-R1	1964	TGCTGGTGTTAATGGCTTA	896
	GIAV9-F2	1191	GTTACTTGAATAGGAGCGATAG	
	GIAV9-R2	1870	AACTTGGAGAGTGTTGACAA	680

Abbreviations: SINV1 GD, Solenopsis invicta virus 1-GD; SINV4 GD, Solenopsis invicta virus 4-GD; GPAV1, Guangdong Polycipiviridae ant virus 1; GDAV2-3, Guangdong Dicistroviridae ant virus 2-3; GIAV4-9, Guangdong Iflaviridae ant virus 4-9; GPAV10, Guangdong Parvoviridae ant virus 10; GAV11-15, Guangdong ant virus 11-15.

1 The position of primers referred to the complete sequences obtained from RNA-seq in this study.

2 UPM: Universal Primer A Mix provided by 5’/3’ RACE kits (TaKaRa, Dalian, China).

3 UPM short: Universal Primer short provided by 5’/3’ RACE kits (TaKaRa, Dalian, China).

Supplementary Table S3. The sequence identity within the SINV4 GD, GPAV1

	Virus name	1	2	3	4	5	6	7	8	9	10	11	12	13
1	NC039236/Solenopsis invicta virus 2/USA		44	43.9	56.3	43.9	44.4	35.4	34.6	32.5	44	34.3	43.6	34.2
2	NC035450/Lasius neglectus virus 1/United Kingdom	44.3		52.3	45.6	65.7	52.2	35.6	33.8	31.8	60.6	33	51.9	33.6
3	NC035455/Solenopsis invicta virus 4/USA	44.8	63		45.9	52.1	68.2	37.3	36.6	34	51.6	35.4	90.8	36.8
4	NC035456/Lasius niger virus 1/United Kingdom	60.2	45.8	42.3		46.3	45.5	43.3	42.8	41	46.2	43.8	45.7	41.8
5	NC035457/Myrmica scabrinodis virus 1/United Kingdom	44.3	86.3	60.9	46.1		52.3	35.5	33.8	32.4	60.2	33	52.3	33.8
6	NC032978/Shuangao insect virus 8/China	43	62.7	79.3	42.3	61.9		36.4	34.9	32.3	51.5	33.6	68	34.3
7	NC033152/Hubei picorna-like virus 81/China	9.4	7.8	8.7	8.5	8.5	9.2		34.8	32.7	36.2	33	37.6	34.9
8	NC032222/Hubei picorna-like virus 82/China	21.9	23.9	23.6	22.8	23.4	23.1	6.5		32.7	33.9	32.9	36.5	33.3
9	MW314658/Lasius neglectus virus 2/Spain	9.7	13.4	12.6	10.9	13	13.1	8.4	9.8		32.8	32	34.3	31.7
10	MZ679315/Polycipiviridae sp./China	45.1	73.1	58.9	46.5	74.1	59.7	8.5	22.6	11.3		33.1	51.7	35
11	PP104791/GPAV1/China	49.2	77.1	66	48.7	78.2	66	10	20.8	12.3	99.5		35.8	32
12	PP104790/SINV4-GD/China	44.6	62.3	98.5	42.1	60.4	79.3	8.8	23.6	12.4	58.6	66		36.6
13	MN832456/Lycopersicon esculentum picorn-like virus/China	23.4	23.5	24.9	25.5	23.8	22.5	10.7	17.7	11.5	24.4	24,5	24.4	

Percent similarity of the complete nucleotide (upper triangle) and amino acid sequences of RdRp (lower triangle) levels calculated via the ClustalW method implemented in MegAlign.

* SINV4 GD, Solenopsis invicta virus 4-GD; GPAV1, Guangdong Polycipiviridae ant virus 1.

Supplementary Table S4. Sequence identity within the SINV1 GD, GDAV2-3, and GAV11-12

		1	2	3	4	5	6	7	8	9	10	11	12	13	14	15	16	17	18	19	20	21	22	23	24
1	NC001834/Drosophila C virus/Australia		36.7	39	35.1	32.9	33.3	33.6	34.2	60.4	36.2	39.3	40.4	32.8	39.1	35.8	35.8	37	62.6	58.5	34.9	34.7	38.6	38.3	37.3
2	NC001874/Rhopalosiphum padi virus/USA	34.9		33.9	32.2	31.9	32	32.2	33.1	35.3	45.4	33.7	34.2	31	33.2	32.9	30.1	32.9	36.5	37.2	29.6	33.5	33	36.8	35.1
3	NC002548/Acute bee paralysis virus/South Africa	40.1	32.6		36.4	32.4	32.3	32.7	33.1	38.2	34	69.4	55.8	31.8	68.3	36.6	32.1	37.3	39.3	39.8	30.3	34.7	52.2	37.3	37.6
4	NC003005/Taura syndrome virus/France	36.2	27.4	40.1		30.5	30.8	31.2	31.9	35.8	31.4	35.8	36.8	30.7	36.3	59.6	29	34	36.2	37.3	28.2	32.4	35.4	35.9	34.1
5	NC003779/Plautia stali intestine virus/Japan	31.6	31.3	31.1	31.3		41.4	40.2	43.9	32	31.8	32.2	32.6	37	31.2	31	31.3	30.3	32.7	33.4	30	40.7	31.6	45.1	43
6	NC003782/Himetobi P virus/Japan	31.1	31.1	30.9	31.9	43.7		41.2	42.4	31.9	30.9	32	33.2	37.2	31.7	31.4	31.4	31.9	33.4	32.7	29.2	43.4	32	45.6	45.2
7	NC003784/Black queen cell virus/South Africa	31.7	28.2	32	28.6	39.4	46.5		42.6	32.7	32.4	32	32.6	37.2	32.4	30.7	32.6	31.1	33.3	34	30.4	42.5	31.8	45.3	43.6
8	NC003783/Triatoma virus/Argentina	29.6	26.1	34.9	29.2	39.8	45.6	44.5		33.8	31.1	32.6	33.9	37.1	33	32.5	33.1	31.5	34	34.9	31.5	42.6	32.7	47.2	44
9	NC003924/Cricket paralysis virus/USA	66.7	33.2	40.6	38.5	29.5	30.2	29.1	30.4		35.5	38.6	39.9	32.6	39	35.1	34.2	37.5	59.5	54.6	33	33.8	37.9	37.4	36.4
10	NC004365/Aphid lethal paralysis virus/The Netherlands	34.5	51.5	33.8	27.3	28.1	29.5	27.8	24.3	32.2		33.2	34.2	30.7	33.3	31.3	30.1	32.9	36.8	37.1	28.5	32.1	32.9	34.1	34.7
11	NC004807/Kashmir bee virus/USA	39.2	31.5	83.3	38.8	31.9	31.7	31.8	34.1	40.3	32.7		55.4	30.8	76.4	35.7	31.7	37.1	38.9	40	30.5	33.7	52.1	37.1	36.3
12	NC006559/ Solenopsis invicta virus 1/USA	41.6	32.5	58.8	38.5	32.1	32.9	31.8	31.9	42.2	34.9	58.6		31.7	54.9	36.4	31.4	38	39.5	44.7	30.3	34.6	94.2	39.5	35.1
13	NC008029/Homalodisca coagulata virus-1/USA	33.4	30	36.1	32.4	37.2	38.8	38.5	38.6	32	29.9	36.6	36		30.8	29.5	29.2	29.7	32.6	33.8	28.2	37.3	30.7	39.9	37
14	NC009025/Israel acute paralysis virus/Israel	38.5	32.6	83.3	38.5	31.9	31.5	31.7	33.6	40.6	33.2	91.3	57.5	36.4		36.6	31.3	37.5	39.8	40.7	30	33.8	52.3	37.2	36.3
15	NC014793/Mud crab dicistrovirus/China	34.4	28.2	38.5	76	27.6	30.5	29	27.4	36.2	25.1	36.9	36.6	31.8	36.4		29.8	34.5	37.3	37.2	29	32.9	35.4	36.5	34
16	PP104800/GIAV6/China	9.7	9.4	12.8	8.2	10.3	9.7	8.5	12.9	9.1	7.8	12.5	13.7	12.1	12.6	10		28.9	34	34.9	34.5	34.9	30.7	35.8	35.6
17	NC032249/Beihai picorna-like virus 70/China	41.8	32.6	46	39.6	30.1	34.7	32.1	32.9	41.6	34.4	45.1	47.9	34.6	44.1	39.3	12.4		38	38.8	28.2	32.1	36.3	33.7	36
18	PP104803/GDAV2/China	63.4	35.3	39.5	35.9	31.4	29	29.3	28.2	61.4	34.1	38.1	40.4	32.1	39.2	35.3	10.7	41.4		61	33.1	34.7	39.5	38.2	34.9
19	KX883903/Beihai picorna-like virus 76/China	71.7	37.1	42.6	40.3	36.6	34.2	35.5	31.1	66.6	38.2	41	46.7	36.2	42.6	39.1	10.7	45.3	67.8		32.8	36.5	39.7	38.1	45.2
20	PP104794/GDAV3/China	10.9	8.7	8.6	8.5	9.1	8.3	8	9	11.4	6.2	7.8	6.4	7.6	7	7.4	7	8.4	9.2	10.7		32.2	30.1	33	33.5
21	NC033437/Wuhan arthropod virus/China	29.9	29.4	33.7	28.5	37.2	51.2	48.2	42.2	29.6	27.1	31.3	30.2	41.7	32.6	29	11.7	35.6	29.7	34.5	9.8		33.9	69.1	69.8
22	PP104795/SINV 1-GD/China	41.9	32.2	58.8	39	31.9	33.2	31.5	31.6	41.7	34.1	58.3	99	36.3	57.2	37.1	13.3	47.7	38.8	45.3	7	30.2		37.4	35
23	PP104805/GAV11/China	35.3	31.6	37.7	35.3	45.5	54	51.7	43.4	34.8	27.5	36.7	35.2	44.4	37.1	34.3	9.9	38	34.2	37.6	10	73.5	33.2		81.1
24	MZ394716/Dicistroviridae sp 1/Japan	32.3	31	34	31.5	40.4	51	48.3	42.5	30.3	25.8	32.2	31.2	40.8	33.8	30.7	9.4	34.9	31	36.1	9.6	73.3	31.2	92.5	

Percent similarity of the complete nucleotide (upper triangle) and amino acid sequences of RdRp (lower triangle) levels calculated via the ClustalW method implemented in MegAlign.

* SINV1 GD, Solenopsis invicta virus 1-GD; GDAV2, Guangdong Dicistroviridae ant virus 2; GDAV3, Guangdong Dicistroviridae ant virus 3; GAV11-12, Guangdong ant virus 11-12.

Supplementary Table S5. Sequence identity within GIAV4-9

		1	2	3	4	5	6	7	8	9	10	11	12	13	14	15	16	17	18	19	20	21	22	23	24	25
1	NC078647/Acheta domesticus iflavirus/Sweden		32.8	28.8	28.7	32	34.7	31.1	33	29.3	33.6	31	29.2	32.8	31.2	27.9	27.1	29.1	29.2	34.9	28.5	28.2	29.3	32	32.7	29.9
2	NC 002066/Sacbrood virus complete genom/UK	26.3		34.1	34.5	38.4	38.9	37.7	37.9	33.3	38	35.8	44.7	37.4	36.6	27.6	28.3	33	31	39.2	32.9	31.5	31.7	37.1	37.7	34.5
3	NC 003113/Perina nuda virus/China Taiwan	23.2	28.2		82.4	34.5	33	34.8	35.6	51.6	34.9	31.5	30.9	32.5	32.7	29.1	28	29.7	31.9	36.2	30	31.9	30.2	35.2	35.8	31.4
4	NC 005092/Ectropis obliqua picorna-like virus/China Hubei	23.2	29.2	92.3		34.9	33	35.2	35.4	51.3	34.6	32.4	31	32.8	32.9	29	28.2	29.8	31.2	36.1	30.2	30.7	29.7	35.4	35.5	31.3
5	NC 004830/Deformed wing virus/Italy	26.9	35.5	25.5	25.3		41	41	43.9	34.5	43.8	37.9	33.9	40.5	39.4	28.4	29.8	32.2	32.4	48.5	31.3	32.8	30.8	40.9	47.6	34
6	NC 009530/Brevicoryne brassicae picorna-like virus/UK	28.9	32.7	25.2	25.6	42.4		42.4	41.9	33.2	42.1	41.2	34.6	40.2	39.2	28.3	30	34.6	30.6	44.4	33.6	30.4	31.4	41.6	41.8	35.8
7	NC 014137/Slow bee paralysis virus/UK	22.9	30	25.1	25.2	39.5	36.4		42.6	34.6	42.8	38.3	33.8	39.8	38.5	30	30	31.5	32	43	31	32.2	31.4	56.8	42.8	34.3
8	NC 023483/Antheraea pernyi iflavirus/China	25.9	31.8	22.2	22.9	46.4	40.1	39.4		34.3	69.7	38.5	33.6	39.5	39.3	29.7	29.9	32.8	32.6	46.4	33.4	33.5	31.7	42.7	45.2	33.6
9	NC 023676/Spodoptera exigua iflavirus 2/South Korea	22.3	28.6	64.2	64.2	25.4	25.6	23.5	23.2		34.6	31.9	31	32.8	32	28.6	28.2	29.9	30.6	35.8	29.7	30.8	29.5	35	34.7	31.9
10	NC 024497/Lymantria dispar iflavirus 1/USA	24.9	29.4	22.6	23.3	46.7	40.2	38.9	82.6	24.8		38.7	33.7	39.7	39.6	29.8	29.7	34.3	31.9	46.4	34.3	32.4	31.9	42.7	44.8	34.4
11	NC 025835/Dinocampus coccinellae paralysis virus/Canada Quebec	26.1	30.9	22.4	21.8	34.3	39.6	31.2	34.1	22.1	32.8		32.6	37.3	36.8	28.7	28.3	31.6	30	41.2	30.3	29.4	29.7	38.2	38.3	32
12	NC 038301/Lygus lineolaris virus/USA	26.6	43	25.7	25.2	33.2	30.7	28.4	31.4	24.2	29.2	27.2		33.7	33.8	27.1	26.4	29.2	27.8	34.9	28.7	28.2	27.8	33.2	34.5	30.8
13	NC 038302/Nilaparvata lugens honeydew virus 1/Japan	24.9	32.2	25.5	27.8	39.5	43.8	35.9	38.9	26.3	39.2	32.5	32.8		38.2	28	29.2	32.5	30.2	42.2	32.5	30.2	30.6	40.2	39.7	34.3
14	NC 040601/Varroa destructor virus 2/Israel	22.9	30.8	23.8	23.8	34.3	36.2	29.2	32.7	25.7	31.8	30.8	29.4	33.1		27.9	27.4	31.1	29.2	40.4	30.8	29.2	29.4	38.6	38.2	33.3
15	PP104792/GIAV9/China	26.5	35.6	28.4	28.9	41.4	38.1	64.9	43.2	28.2	41	33.2	32.4	37.9	33.7		25.1	30	29.3	29.5	27.9	28.2	28.4	29.6	29.4	27.8
16	PP104793/GIAV8/China	24.6	34.6	28	28.1	42.4	37.1	63.6	42.9	27.4	41.3	33.2	30.1	38.5	32.1	79.1		29.8	29.1	29.7	27.9	28.6	29.3	29.6	29.9	27.3
17	PP104798/GIAV6/China	22.3	30.3	24.4	24.8	27.1	28.3	28.8	29.8	22.6	28.5	24.8	28.7	28.3	30	29.3	29		30.3	35.8	34.4	29	32.1	32.5	33.3	29.8
18	PP104799/GIAV4/China	21.3	27.4	23.3	22.6	28.7	26.7	26.5	28.2	23.7	28.2	24.8	26.1	25.1	27.8	28.7	27.7	44		32.9	29.4	60.8	44.9	31.5	32.5	28.8
19	PP104801/GIAV5/China	24.1	38.2	26.3	26.4	48.4	40.7	38.4	48.7	27.9	46.5	35.8	33.2	41.7	37.4	41.4	39.8	25.7	28.4		33.6	32.7	32.9	44.2	58.2	35.7
20	PP104802/GIAV7/China	30.6	31.7	35.6	35	33	37.3	34.5	31.5	33.7	30.5	37.9	30.4	33.7	32.1	31.7	33	27	28.3	31.2		28.3	30	31.7	32.5	29.6
21	NC 003781/Infectious flacherie virus/Japan	19.6	24.1	20.7	20.7	24	24.3	23.3	24.1	20.8	23.9	20.5	22.4	23.2	25.4	25	25	42.9	74.2	26	29.2		43.9	31.8	32.5	29
22	NC 027917/Opsiphanes invirae iflavirus 1/Brazil	21.4	27.1	20.2	20.6	23.9	26.1	22.7	26.5	19.9	26.1	21.7	26.1	25.9	24.3	28.3	26.4	73	44.9	26.7	27.3	40.2		30.7	31.5	29.3
23	NC 031338/Moku virus/USA	25.6	33.1	26.2	26.2	42.6	38.7	58.3	42.4	25	42.8	34.4	28.6	37.8	29.6	68.4	70.3	28.1	28.1	42.3	33.2	22.4	24.7		42.2	33.6
24	NC 031749/King virus/USA	24.5	33.8	24	23.8	49.7	39.7	37	46.5	26.3	46.6	37.8	29.3	38.5	31.3	39.7	39.4	27.5	28.2	57.4	33.8	25.1	25.3	41.1		33.8
25	MH620811/Lampyris noctiluca iflavirus 1/Finland	27.9	32.4	29.4	29.8	29.5	32.2	29.7	31.9	28	31.2	29.3	29.4	28.6	28.5	30.1	29.8	26.1	25.8	30.3	52.4	21.9	23.9	31.1	28.1	

Percent similarity at the OF complete nucleotide (upper triangle) and amino acid sequence of RdRp (lower triangle) levels calculated via the ClustalW method implemented in MegAlign.

*GIAV4-9, Guangdong Iflaviridae ant virus 4-9.

Supplementary Table S6. Comparison between Guangdong and USA RIFA viruses

Family	Species in Guangdong RIFA	Species in USA RIFA	Specific descriptions
Polycipiviridae	Solenopsis invicta virus 4-GD (90.8% compare with USA NC 035455)<br>Guangdong Polycipiviridae ant virus 1	Solenopsis invicta virus 4 (NC_035455)<br>Solenopsis invicta virus 2 (NC_039236, MG676340, EF428566)	Both the USA and Guangdong have *Polycipiviridae* viruses, but only the species *Solenopsis invicta virus 4* were found in both place.
Dicistroviridae	Solenopsis invicta virus 1-GD (94.2% compare with USA NC 006559)<br>Guangdong Dicistroviridae ant virus 2<br>Guangdong Dicistroviridae ant virus 3	Solenopsis invicta virus 1 (NC 006559, AY634314)<br>Solenopsis invicta virus 6 (MH714708, MH714707)<br>Solenopsis invicta virus 9 (MH727526)<br>Solenopsis invicta virus 12 (MH727529)<br>Solenopsis invicta virus13 (MH727530)	Both the USA and Guangdong have *Dicistroviridae* viruses, but only the species *Solenopsis invicta virus 6* were found in both place.
Iflaviridae	Guangdong Iflaviridae ant virus 4<br>Guangdong Iflaviridae ant virus5<br>Guangdong Iflaviridae ant virus 6<br>Guangdong Iflaviridae ant virus 7<br>Guangdong Iflaviridae ant virus 8<br>Guangdong Iflaviridae ant virus 9	Solenopsis invicta virus 11 (MH727528)<br>Solenopsis invicta virus 16 (MT860233)<br>Solenopsis invicta virus 17 (MT860234)<br>King virus (MH778500, MH778501)	Both the USA and Guangdong host viruses from the *Iflaviridae* family; however, no identical viral species have been found in both locations.
Parvoviridae	Guangdong Parvoviridae ant virus 10	Nd	Only Guangdong
Solinviviridae	Nd	Solenopsis invicta virus 3 (NC_012531, MF797911, GU017972, FJ528584)<br>Nylanderia fulva virus 1 (MG696804, MG696805, MG696806)	Only USA
Totiviridae	Nd	Solenopsis midden virus (MH727531)	Only USA
Nyamiviridae	Nd	Solenopsis invicta virus 15 (MT860232)	Only USA
Phenuiviridae	Nd	Solenopsis invicta virus 14 (MT860240, MT860241, MT860242)	Only USA
unclassified viruses	Guangdong ant virus 11<br>Guangdong ant virus 12<br>Guangdong ant virus 13<br>Guangdong ant virus 14<br>Guangdong ant virus 15	Solenopsis invicta virus 5 (MF593921)<br>Solenopsis invicta virus 7 (MH719200)<br>Solenopsis invicta virus 10 (MH727527)	Both the USA and Guangdong have unclassified viruses, no identical viral species have been found in both locations.

Nd: No data
